# Self-assembly of pyrene-appended glucono gelators: spacer regulated morphological change and inversion of circularly polarized luminescence†

**DOI:** 10.1039/c9ra10315e

**Published:** 2020-02-13

**Authors:** Zongwen Liu, Yuqian Jiang, Jian Jiang, Chenhuan Yuan, Decai Wang, Minghua Liu

**Affiliations:** College of Biotechnology and Pharmaceutical Engineering, Nanjing Tech University Nanjing 211816 Jiangsu P. R. China dcwang998@126.com; CAS Center for Excellence in Nanoscience, CAS Key Laboratory of Nanosystem and Hierarchical Fabrication, Division of Nanophotonics, National Center for Nanoscience and Technology (NCNST) No. 11 ZhongGuanCun BeiYiTiao Beijing 100190 P. R. China jiangj@nanoctr.cn liumh@iccas.ac.cn; Beijing National Laboratory for Molecular Science, CAS Key Laboratory of Colloid Interface and Chemical Thermodynamics, Institute of Chemistry, Chinese Academy of Sciences No. 2 ZhongGuanCun BeiYiJie Beijing 100190 P. R. China

## Abstract

Pyrene-appended glucono gelators with different spacer lengths (two and four methylene units) were designed and found to form supramolecular gels in organic aqueous solvents. The shorter spacer gelator 1 was prone to self-assemble into nanotubes due to well stacking multi-bilayer unit, while gelator 2 with the longer spacer formed nanofibers due to the relatively disordered packing structure. Both of the gels showed supramolecular chirality as well as circularly polarized luminescence (CPL) due to the chirality transfer from the glucose moiety to the assembly. Interestingly, the CD and CPL signals were opposite for the two gels. It was suggested that the packing of the pyrene unit in the gels were different due to the spacer and resulted in the inversed chiroptical properties. The work provided a deeper understanding of the origin of the supramolecular chirality and furthers the design of the CPL materials.

## Abstract 2

Pyrene-appended glucono gelators with different spacer lengths (two and four methylene units) were designed and found to form supramolecular gels in organic aqueous solvents.

## Graphical abstract

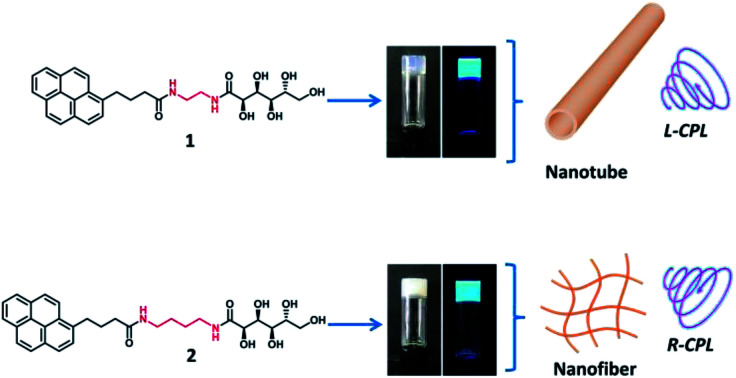

Self-assembly offers an efficient method to construct supramolecular gels, where small gelator molecules self-assembled into nanostructures and immobilized solvents *via* non-covalent interactions including hydrophobic interaction, hydrogen bonding, π–π stacking, electrostatic interaction, van der Waals forces and charge–transfer interactions.^1–5^ Interestingly, many of the chiral molecules easily form supramolecular gels meaning that the gels can be applied in chiral recognition, chiral separation, asymmetric catalysis and chiroptical functional materials.^6–16^

Circularly polarized luminescence (CPL) is a unique function that pertains to chiral systems, which emit different left- and right-handed circularly polarized light and have attracted great interest in recent years.^16–22^ By combining the chiral units and fluorophores, many of the chiral molecular self-assembly systems have been developed and efficient chiroptical CPL have been realized.^18–22^

In general, molecular systems show only one direction of CPL, this is determined by the molecular chirality of the chiral moiety. However, at a supramolecular level, supramolecular chirality can be controllably changed *via* regulating the self-assembly process or slightly modifying the molecules while maintaining the molecular chirality. For example, DNA could have right-handed B-DNA and left-handed Z-DNA was formed while originating from the same molecular chirality of d-sugar.^23^ In an artificial system, supramolecular chirality was also observed to be reversed by external stimulus. For example, the opposite CD spectra can be achieved through changing solvent, temperature, light irradiation, and addition of metal ions or achiral dopants.^24–28^ However, there were only limited cases that realize the inversion of CPL in supramolecular gels by tuning the self-assembly process.^29–32^ It still remains unknown why such chirality inversion could happen in supramolecular systems.

Here we designed two pyrene based derivatives (1–2, Fig. 1), in which a pyrene core served as an emission moiety, and a glucono group served as chiral source. The two parts were covalently connected *via* two amide bonds with different lengths of alkyl spacers. These molecules (1–2) can form supramolecular gels in the organic aqueous media. The self-assembled behaviour and luminescent property of the gel could be tuned dependent on the solvents and length of alkyl spacer chains, the nanotubes and nanofibers together with opposite CD and CPL signals were observed based on the different length of space alkyl chain in the gelator, as illustrated in Fig. 1.

**Fig. 1:**
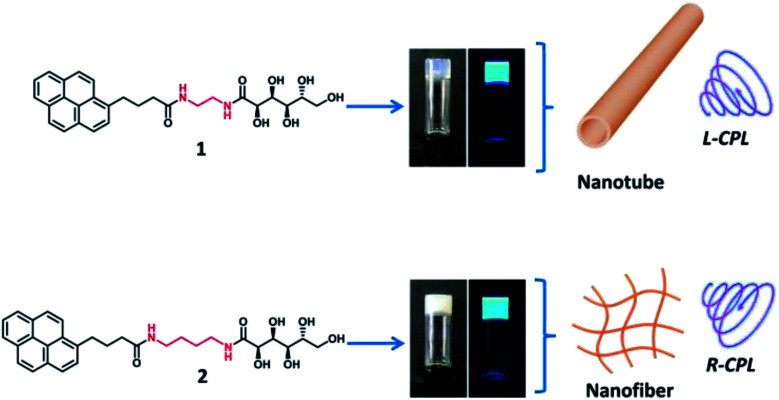
Molecular structure of gelator 1–2 and photographs of formed gel in EtOH–H_2_O mixture solution (EtOH : H_2_O = 1 : 2) under natural light and UV light (365 nm). The gelator 1 self-assembled into nanotube with left-handed CPL, while gelator 2 self-assembled into nanofibers with right-handed CPL.

4-(1-Pyrenyl)butyric acid methyl ester was employed as staring material, which was first reacted with ethylenediamine and 1,4-butanediamine respectively, the intermediate was separated and purified by gel column chromatography, and the desired gelator 1–2 was obtained after the intermediates were further reacted with glucono δ-lactone (ESI†).

The gelation behaviour of molecule 1–2 was tested in various solvents at a standard concentration with 1% (w/v). The results were summarized in Table S1,† both gelator 1 and gelator 2 were insoluble in a series of polar and nonpolar organic solvents including petroleum ether, dichloromethane, *n*-hexane, cyclohexane, acetone, ethyl acetate, THF, acetonitrile, methanol and ethanol (Table S1,† entry 1–10), and complex 1–2 were soluble in DMF and DMSO (Table S1,† entry 11 and 12). To our delicious, the compound 1–2 can form gel in the organic-water mixture solutions, including THF–H_2_O, DMSO–H_2_O and EtOH–H_2_O. In the case of ethanol water mixture solvent, the molecule 1 and 2 was first dissolved in ethanol–H_2_O (1 : 1) mixture solution (Table S1,† entry 13). Further improving the content of water, the gel was formed (Table S1,† entry 14–16). Especially, when the volume ratio of EtOH : H_2_O equal to 1 : 2, gelator 1 formed nearly transparent gel with critical gelation concentration (CGC) about 3 mg mL^−1^, while gelator 2 formed translucent gel with the CGC about 5 mg mL^−1^ (Fig. 1, photographs). In addition, the white gels were observed in the pure water (Table S1,† entry 17). Furthermore, the complex 1–2 exhibited similar gel behaviour in THF and DMSO aqueous solution as in ethanol–water solution (Table S1,† entry 18–22).

The morphology of 1 and 2 based gel in ethanol aqueous solution was first investigated (the gels were named as gel_1_ and gel_2_ corresponding to gelator 1 and gelator 2). It was found that the morphology of gel_1_ have obvious solvent dependent behaviour, scanning electron microscope (SEM) images shown that gel_1_ underwent change from nanofibers to nanorods and further to amorphous nanosheets in ethanol aqueous solution when gradually increasing the water content (Fig. S1†). It should be noted that uniform nanofibers were observed when gel_1_ formed in 1 : 2 of ethanol : H_2_O mixture solvent, the diameter distribution of nanofibers is extremely narrow (15 nm ± 1 nm), the length of nanofibers reached tens to hundred micrometers (Fig. 2a and S2†). TEM image indicated that these nanofibers were actually nanotubes, in which the diameter of nanotube was about 15 nm and the wall thickness of nanotube was around 2.5–2.7 nm (Fig. 2b and S2†). AFM image further verified that the height of the nanotube was around 15 nm (Fig. 2c and S3†). In the case of gel_2_, the nanofibers with the diameter around a few nanometers to tens of nanometers were observed in ethanol aqueous solution (Fig. S4†). Also, the size distribution of nanofibers was relatively uniform at 1 : 2 of ethanol water solution (Fig. 2d). TEM and AFM images further proved that gel_2_ was nanofiber structure with the diameter of nanofiber around 5–10 nm (Fig. 2e, f and S5†). These results indicated that assembly behaviour of gelator 1 and 2 formed well defined nanostructures under 1 : 2 ethanol water mixed solvent.

**Fig. 2:**
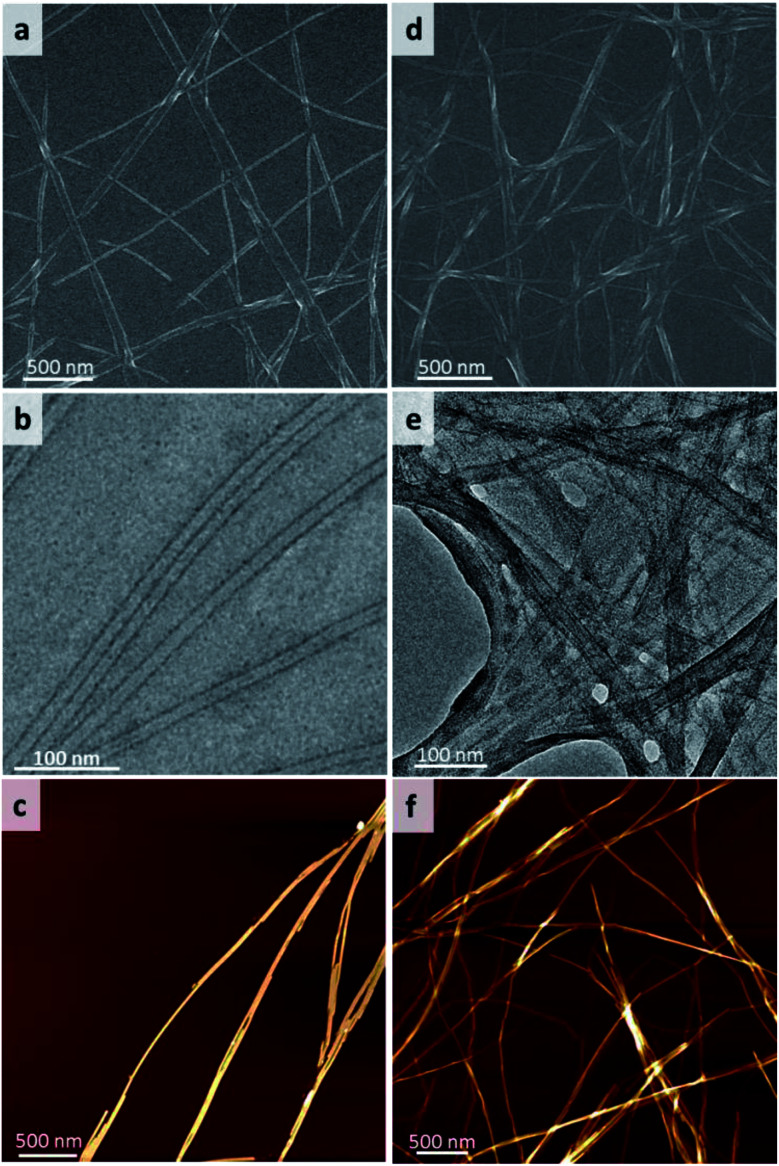
SEM images of (a) gel_1_ and (d) gel_2_, TEM images of (b) gel_1_, (e) gel_2_ and AFM of images of (c) gel_1_ and (f) gel_2_. The gels were formed in mixed solvent of ethanol and water (EtOH : H_2_O = 1 : 2).

UV-vis spectra showed four main peaks at 266 nm, 277 nm, 328 and 345 nm of gelator 1 in DMSO dilute solution (Fig. 3a, black line, 1 × 10^−5^M), which can be assigned to the un-aggregated form of pyrene units (Fig. S6,† SEM and TEM of gelator 1 and 2 in DMSO indicated no assembly happened). The absorption peaks are blue shifted to 265 nm, 275 nm, 326 nm and 343 nm for gelator 1 in gel state (Fig. 3a, red line, gel in 1 : 2 of ethanol : water). The blue-shifted bands were also observed in gel_2_ in comparison with 2 in DMSO diluted solution (Fig. 3b), these results suggested that in both cases, the gelator 1–2 prone to form H-aggregates in the gel state. Furthermore, it was found that the UV-vis spectra of gel_1_ and gel_2_ in different volume ratio of ethanol and water were nearly same as in 1 : 2 of ethanol : water solution (Fig. S7†).

**Fig. 3:**
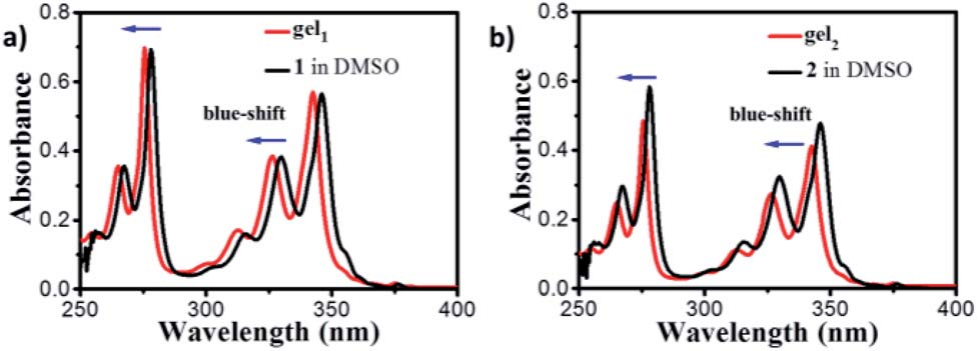
(a) UV-vis spectra of gelator 1 in DMSO (black line) and gel_1_ in 1 : 2 of ethanol : water mixed solvent (red line). (b) UV-vis spectra of gelator 2 in DMSO (black line) and gel_2_ in 1 : 2 of ethanol : water mixed solvent (red line).

For exploring the emission properties of 1–2 gels, the fluorescence spectra were measured. In the diluted DMSO solution, two emission peaks at 380 nm and 396 nm ascribed to the monomer emission together with a very weak peak at 480 nm belong to excimer emission were observed for gelator 1 (Fig. 4a, purple line). In the gel_1_, the monomer emission peak decreased and excimer peak increased significantly, and the strong excimer emission peak at 442 nm was observed for the gel in 1 : 2 of ethanol : water mixture solution (Fig. 4a, black line). This phenomenon was also observed in the case of gel_2_, and found that the strong excimer peak at and 470 nm was observed (Fig. 4b, black line, gel in 1 : 2 of ethanol : water). The fluorescence quantum yields of gel_1_ and gel_2_ was about 28.2% and 9.4% respectively. Obviously, these results indicated that both gel_1_ and gel_2_ have strong π–π interaction between pyrene moieties.

**Fig. 4:**
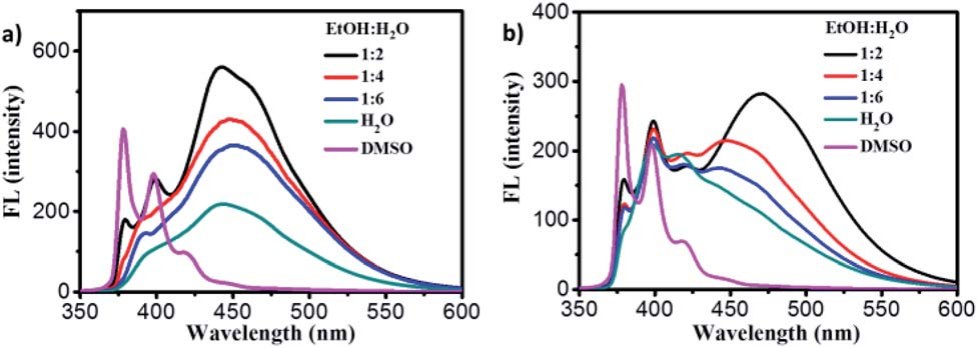
a) Fluorescence spectra of gelator 1 in DMSO (purple line) and gel_1_ in EtOH/H_2_O mixture solution. (b) Fluorescence spectra of gelator 2 in DMSO (purple line) and gel_2_ in EtOH/H_2_O mixture solution.

Since the glucono group is chiral, the chiroptical properties of gels were further explored. There revealed several interesting features. First, when the gelator molecules 1–2 were dissolved into DMSO solution as a molecular state, no CD spectra can be detected in the absorption band of pyrene (Fig. S8†). However, obvious CD signals were observed when compounds 1–2 formed gels in mixed ethanol and water (1 : 2) solvent. The negative CD signal of gel_1_ with the peaks at 294 nm, 366 nm and 382 nm were observed (Fig. 5a), which were consistency of corresponding to the UV-vis spectra of gel_1_, indicated that pyrene segments oriented in the chiral manner in the gel state. In the case of gel_2_, to our surprise, the positive CD signal with the peak at 290 nm, 344 nm and 367 nm were observed. It should be noted that gel_2_ presented opposite CD signal in comparison with gel_1_ in the longer wavelength regions. These results indicated that chiral packing manners of chromophore pyrene units in the gel state could be different, where the gelator spacer may regulate the supramolecular chirality of resulted gel assemblies. For quantitatively discuss comparison the supramolecular chirality, the dissymmetry factor of ECD, *g*_CD_ value were found to be 9 × 10^−4^ and 1.4 × 10^−4^ for gel_1_ and gel_2_ respectively, indicated that gel_1_ with more uniform nanotube structure gave a stronger *g*_CD_. The CD signal based on the pyrene moiety was due to the chirality transfer from the glucose. Since in gel_1_, the distance between the pyrene and chiral unit is shorter, it is more efficient to transfer the chirality, thus leading to a larger *g*_CD_ In addition, the CD spectra of gel_1_ and gel_2_ under different volume ratio of ethanol and water were also measured, and found that all of gel_1_ samples under different ethanol aqueous solution present negative CD, all of gel_2_ samples under different ethanol aqueous solution present positive CD (Fig. S9†), proved that the supramolecular chirality of both gel_1_ and gel_2_ was stable toward ethanol aqueous solvents.

**Fig. 5:**
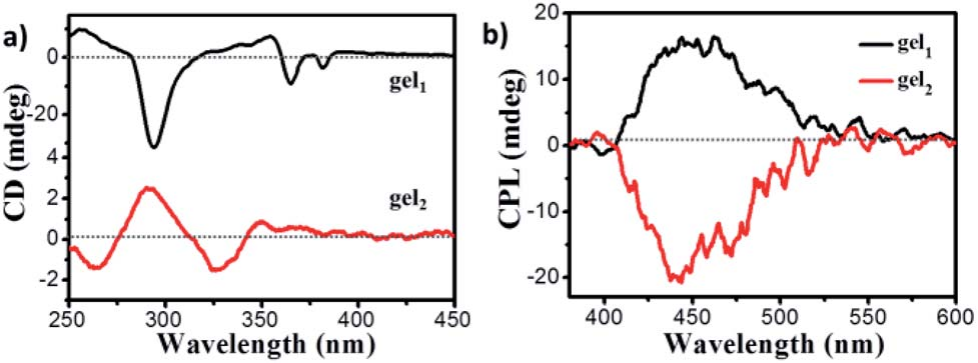
(a) CD spectra of gel_1_ (black line) and gel_2_ (red line); (b) CPL of gel_1_ (black line) and gel_2_ (red line) (gelled in EtOH/H_2_O mixture solution, EtOH : H_2_O = 1 : 2), excited at 320 nm.

Since the gel is fluorescent, the CPL spectra of gels were further investigated. It was found that both of gels exhibited CPL signals. The gel_1_ exhibited CPL signal with a maximum emission peak at 445 nm, which was coincided with the fluorescent excimer emission peaks (Fig. 5b). However, no obvious CPL can be detected when gelator 1 dissolved in DMSO (Fig. S10†), confirmed that CPL was originated from the supramolecular chiral aggregation of fluorescent pyrene units in the gel. The luminescence dissymmetry factor, *g*_lum_ = 2 × (*I*_L_ − *I*_R_)/(*I*_L_ + *I*_R_) was used to characterize CPL performance, where *I*_L_ and *I*_R_ are the luminescence intensities of right and left circularly polarized light respectively. The *g*_lum_ of gel_1_ is about 2.5 × 10^−3^. As expected, gel_2_ exhibited a nearly completely opposite CPL signal in comparison to gel_1_, the *g*_lum_ of which is about −3.3 × 10^−3^. Therefore, it was confirmed that supramolecular chiral alignment of pyrene fragments in the gel is able to emit the CPL, while changing the length of space in the gelator can inverse CPL. In addition, the CPL of gel_1_ and gel_2_ under different volume ratio of ethanol and water were also investigated, and found that the highest *g*_lum_ was found at 1 : 2 ethanol and water solvent both for gel_1_ and gel_2_ (Fig. S11†), indicated that ordered assembly architectures gave a higher CPL intensity.

X-ray diffraction (XRD) was employed for exploring the molecular pacing in xerogels. As shown in Fig. 6a, gel_1_ showed the peaks with *d*-spacing of 2.7, 1.4 and 0.90 nm based on the Braggs eqn, indicating a lamellar packing structure according to the ratio of 1, 1/2 and 1/3. The *d* space of lamellar structure is about 2.7 nm, which is close to the wall thickness of nanotube observed by TEM. In the case of the gel_2_, the gelators also self-assembled into the lamellar structure with *d* spacing of 2.6 nm according to the diffraction peaks, suggesting that nanofibers consist of gel_2_ was about two to multi lamellar thickness.

**Fig. 6:**
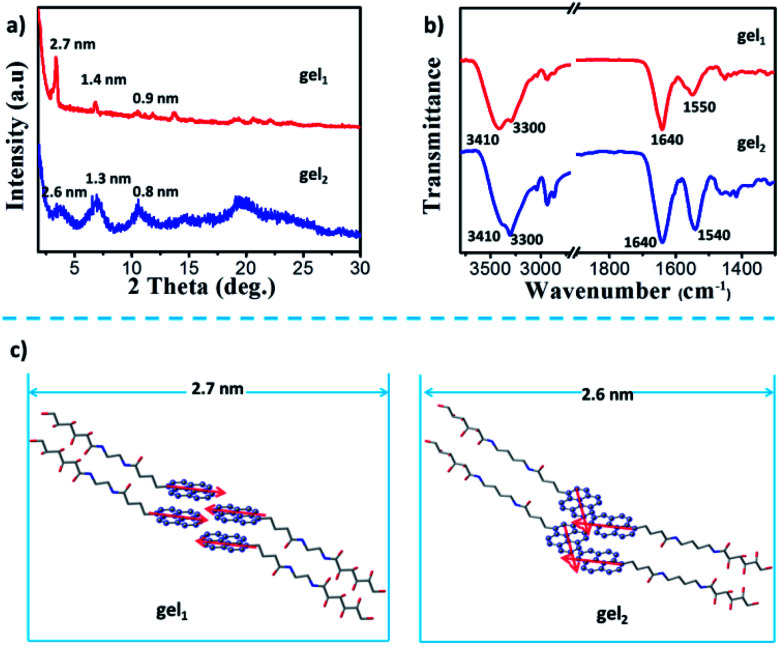
a) X-ray diffraction patterns of the xerogels of gel_1_ and gel_2_. (b) FTIR spectra of xerogels of gel_1_ and gel_2_. (c) The probable packing manner of gel_1_ and gel_2_. The pyrene segments in gel_1_ were parallel packing, gave left-handed CPL; while the pyrene segments in gel_2_ were overlapped packing, gave right-handed CPL.

FT-IR spectrum was employed to provide the interaction modes in the gels. Two strong vibrations were observed at 3410 and 3300 cm^−1^, which could be assigned to O–H and N–H vibrations, respectively. These two bands were from the glucone unit and the amide band, respectively. From the wavenumbers of the vibrations, it is clear that these gelators molecules formed H-bond between the glucone and amide groups. In addition, in both of gel state, the band at 1640 cm^−1^ were observed, which was assigned to the vibration of amide I. However, the different in the position and hydrogen bonding interaction showed characteristic peaks for a hydrogen bonded amide II, the stretching bonds at 1550 cm^−1^ was belonged to gel_1_, and gel_2_ shown the vibration band at 1540 cm^−1^ (Fig. 6b), indicated the hydrogen-bonding interaction between gelators in gel_2_ was weaker than that in gel_1_.

Based on these data, a possible packing model of gel_1_ and gel_2_ was proposed. In both of the gel systems, the molecules packed in a basic bilayer mode, like many of the amphiphiles. Here, the glucone group are in the outside while the pyrene packed inside the bilayer. Such bilayers were stabilized by the intermolecular H-bond between amide and the glucono unit, as shown in the FT-IT spectra. Furthermore, the strong excimer peak was observed, indicating that π–π interactions existed in both gel systems. It should be noted the π–π conjugated degree between pyrene units of gel_2_ may be different, which is regulated by the spacers. It should be noted that gelator 2 has a longer spacer length with four methylene units, while gelator 1 has only two methylene units. However, the *d* spacer from the XRD is longer for gel_1_ (2.7 nm), while shorter for gel_2_. This indicated that in gel_2_, the molecules are more tilted oriented. A possible orientation could be illustrated as in Fig. 6c. In the case of gel_1_, pyrene packed parallel to each other and the bilayer showed ordered structures. Due to the ordered packing, the bilayer rolled into nanotube and showed a stronger excimer emission. Due to the well-packing in an H-aggregate, the excimer appeared in a relatively shorter wavelength. In the gel_2_, due to the longer spacer and flexibility, the pyrene packed relatively disordered. It seems that the pyrene can interpenetrate between each layers and caused partial overlap stacking of the pyrene unit, which can be seen from the Cotton effect in the case of gel_2_, where a split existed around 250–300 nm, belonging to the shorter axis of the pyrene. Thus, in gel_1_, the dipole moment of the pyrene is parallel, while in gel_2_, those of the dipole moments constituted a larger angle, as indicated in Fig. 4c. According the exciton theory, these two alignments would cause the opposite CD signals. Thus, we observed the opposited CD signals in gel_1_ and gel_2_. In the excited state, it seemed that there is no big change of the conformation, thus, we observed also the opposite CPL. Thus, through the spacer regulation, we can realized the inversion of CD and CPL signals, which provided a deep insight into the origin of the supramolecular chirality and regulating method.

In conclusion, two pyrene based gelators were designed and found to form gels in the mixed solvents of water and ethanol. Due to the hydrogen bond between amide and glucone and the π–π stacking of the pyrene, lamellar structures were formed as a basic unit. The shorted spacer of gel_1_ caused the well-defined π–π stacking of the pyrene and rolled into nanotube. In gel_2_, a well-packed bilayer unit and relatively disordered packing of pyrene lead to the nanofiber formation. The molecular chirality of the glucone unit can transfer to the assemblies that lead to the CD and CPL of the nanotube and nanofiber. Interestingly, the nanotube and the nanofiber showed the opposite CD and CPL signals, which was suggested to be due to the different packing of the pyrene units in the lamellar structures. Thus, the work provided a new understanding of the origin of the supramolecular chirality and a way to regulate the chiral self-assembly and the chiroptical *via* spacer length.

## Conflicts of interest

The authors declare no conflict of interest.

## Supplementary Material

RA-010-C9RA10315E-s001
