# Supplementary material for: Self-assembly of pyrene-appended glucono gelators: spacer regulated morphological change and inversion of circularly polarized luminescence
Source: RSC Adv. 2020 Feb 13;10(12):6772–6. doi: 10.1039/c9ra10315e (PMC9049720; doi:10.1039/c9ra10315e)
Supplement: RA-010-C9RA10315E-s001 [file RA-010-C9RA10315E-s001.pdf]

Electronic Supplementary Material (ESI) for RSC Advances.
This journal is © The Royal Society of Chemistry 2020

Supporting Information

Self-assembly of Pyrene-appended Glucono Gelators: Spacer Regulated Morphological Change and Inversion of Circularly Polarized Luminescence

Zongwen Liu, [ab] Yuqian Jiang, [b] Jian Jiang,*[b] Chenhuan Yuan,[c] Decai Wang*[a] and Minghua Liu*[bc]

[a]College of Biotechnology and Pharmaceutical Engineering, Nanjing Tech University, Nanjing 211816, Jiangsu, P. R. China.

[b]CAS Key Laboratory of Nanosystem and Hierarchical Fabrication, CAS Center for Excellence in Nanoscience, Nanophotonics Research Division, National Center for Nanoscience and Technology (NCNST) No.11 ZhongGuanCun BeiYiTiao, 100190 Beijing, P.R. China.

[c]Beijing National Laboratory for Molecular Science, CAS Key Laboratory of Colloid, Interface and Chemical Thermodynamics, Institute of Chemistry, Chinese Academy of Sciences, No.2 ZhongGuanCun BeiYiJie, 100190, Beijing P. R. China.

Table of Contents

S1. Characterization and synthetic measures..1

S2. Supplementary Figures...5

S1. Characterization and synthetic measures

Characterization: The ^{1}H NMR spectra were recorded on a Bruker Avance III 400 (400 MHz) spectrometer. UV-vis spectra were recorded in quartz cuvettes with 1 cm light path on a SHIMADZU UV-2600 spectrophotometer from 250 nm to 400 nm. CD spectra were obtained using a JASCO J-815 CD spectrophotometer in quartz cuvettes with a 1 mm path length in the range of 250-500 nm. Fluorescence spectra were recorded in quartz cuvettes with 1 mm light path on a Hitachi F-4600 spectrometer. All gels were measured with excitation at 320 nm. The images of SEM were recorded using a Hitachi S-4800 FE-SEM under an accelerating voltage of 10 kV. The crystal structure (XRD) of xerogels was recorded on a Rigaku D/Max-2500 X-ray diffractometer (Japan) with Cu Kα radiation. The scan speed was 4° min^{-1} and scan range from 1 to 30°. Circularly Polarized Luminescence (CPL) measurements were recorded on a JASCO-200 CPL spectrometer.

Preparation of hydrogels and organogels: Adding 5 mg sample into 1 ml pure water, and heat to accelerate dissolving. In order to form hydrogel, cooling it to room temperature after getting a clearly solution. Orgnogels were prepared by dissolving 5mg sample into 1ml ethanol-water solution (ethanol : water=1:2).

Materials: 1-Pyrenebutyric acid, ethylenediamine, 1,4-Diaminbutane and delta-Gluconolactone were purchased from Innochem. All chemical solvents were provided by Beijing Chemicals.

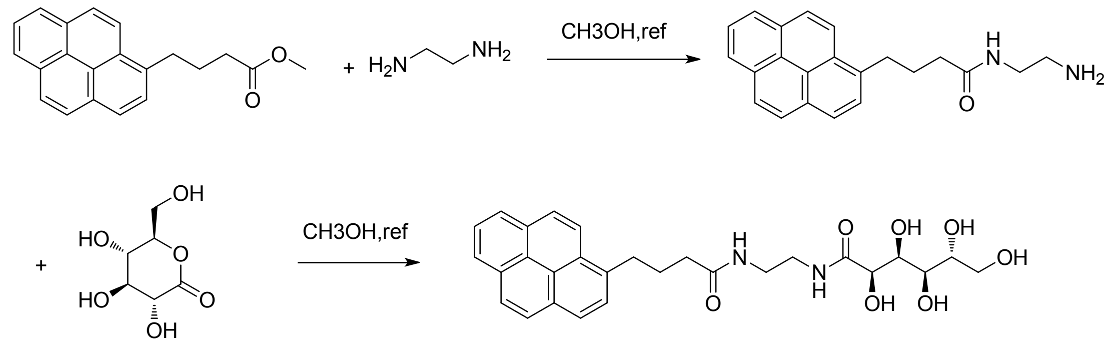

Scheme S1. Synthesis of gelator 1

Synthesis of gelator 1: Firstly, 1-Pyrenebutyric acid and thionyl chloride were dissolved in methanol and reacted for 30 minutes at 0℃，increased the temperature to 70℃ and continued to react for 6 h to yield 4-(1-pyrenyl)butyric acid methyl ester. Then, 4-(1-pyrenyl)butyric acid methyl ester (3.0 g, 10 mmol) was dissolved in methanol (300 ml), ethylenediamine (1.2 g, 20 mmol) was added and the mixture was stirred for 24h at 80℃. The solvent was removed under reduced pressure to obtain 2,3,4,5,6-pentahydroxy-N-(2-(4-(pyren-1-yl)butanamido)ethyl)hexanamide. Finally, The gelator 1 was prepared by 4-(pyren-1-yl) butanamido)ethyl)hexanamide with delta-Gluconolactone (3.56 g, 20 mmol) in methanol and stirred at 80℃ for 12 hours. The solvent was removed under reduced pressure and washed three times with water, then dried under vocuum to give 3.2 g white solid.

^{1}H NMR (400 MHz, DMSO): δ 8.39 (d, *J* = 9.3 Hz, 1H), 8.30 – 8.17 (m, 4H), 8.16 – 8.08 (m, 2H), 8.05 (t, *J* = 7.5 Hz, 1H), 7.94 (d, *J* = 7.7 Hz, 1H), 7.87 (s, 1H), 7.81 (s, 1H), 5.39 (d, *J* = 4.4 Hz, 1H), 4.55 (dd, *J* = 18.0, 3.3 Hz, 2H), 4.46 (d, *J* = 6.9 Hz, 1H), 4.36 (t, *J* = 5.4 Hz, 1H), 4.01 (d, *J* = 3.8 Hz, 1H), 3.95 (d, *J* = 6.4 Hz, 1H),3.66 – 3.27 (m, 6H), 3.19 (s, 4H), 2.24 (t, *J* = 7.1 Hz, 2H), 2.09 – 1.94 (m, 2H).

MALDI-TOF: m/z (%): calcd. for C28H32N2O7 M^+: m/z=508.22; found M^+: m/z=507.9 and $[M+Na]^+$: m/z=530.8

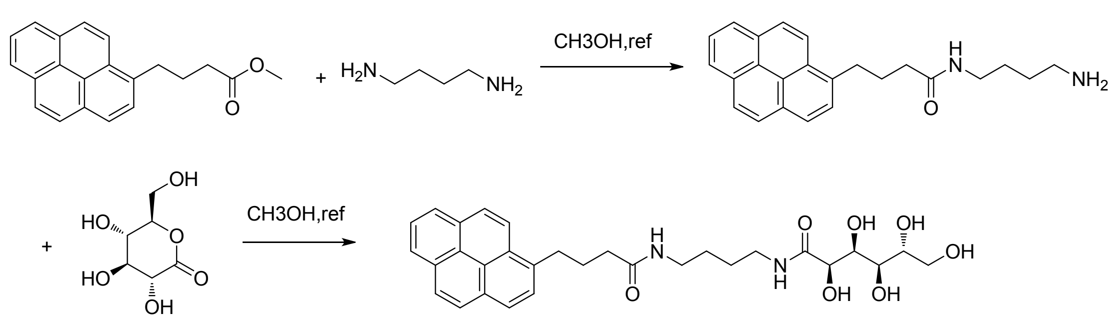

Scheme S2. Synthesis of gelator 2

The synthesis methods of gelator 2 were the similar with gelator 1

^{1}H NMR (400 MHz, DMSO): δ 8.38 (d, *J* = 9.2 Hz, 1H), 8.30 – 8.19 (m, 4H),

8.16 – 8.09 (m, 2H), 8.05 (t, *J* = 7.6 Hz, 1H), 7.93 (d, *J* = 7.7 Hz, 1H), 7.82 (s, 1H), 7.63 (s, 1H), 5.36 (d, *J* = 4.5 Hz, 1H), 4.52 (d, *J* = 26.5 Hz, 2H), 4.40 (d, *J* = 7.1 Hz, 1H), 4.35 (s, 1H), 3.99 (s, 1H), 3.92 (s, 1H), 3.63 – 3.45 (m, 3H), 3.34 – 3.24 (m, 3H), 3.13 – 2.99 (m, 4H), 2.23 (t, *J* = 7.0 Hz, 2H), 2.08 – 1.93 (m, 2H), 1.41 (s, 4H).

MALDI-TOF: m/z (%): calcd. for C30H36N2O7 M^+: m/z=536.25; found M^+: m/z=537.0 and $[M+Na]^+$: m/z=559.0

S2. Supplementary Figures

Table S1. Solvent selection of gelators 1-2

Entry	Solvent	state 1	state 2	CGC **1** (mg/mL)	CGC **2** (mg/mL)
1	petroleum ether	I	I	-	-
2	dichloromethane	I	I	-	-
3	n-hexane	I	I	-	-
4	cyclohexane	I	I	-	-
5	acetone	I	I	-	-
6	ethyl acetate	I	I	-	-
7	THF	I	I	-	-
8	acetonitrile	I	I	-	-
9	methanol	I	I	-	-
10	ethanol	I	I	-	-
11	DMF	S	S	-	-
12	DMSO	S	S	-	-
13	ethanol-H_2O 1-1	S	S	-	-
14	ethanol-H_2O 1-2	G	G	3	5
15	ethanol-H_2O 1-4	G	G	4	5
16	ethanol-H_2O 1-6	G	G	4	6
17	H_2O	G	G	5	5
18	THF-H_2O 1-1	S	S	-	-
19	THF-H_2O 1-2	S	S	-	-
20	THF-H_2O 1-5	G	G	5	5
21	DMSO-H_2O 1-1	G	S	5	-
22	DMSO-H_2O 1-2	G	G	4	6

S:solution, I:insolution, G:gel

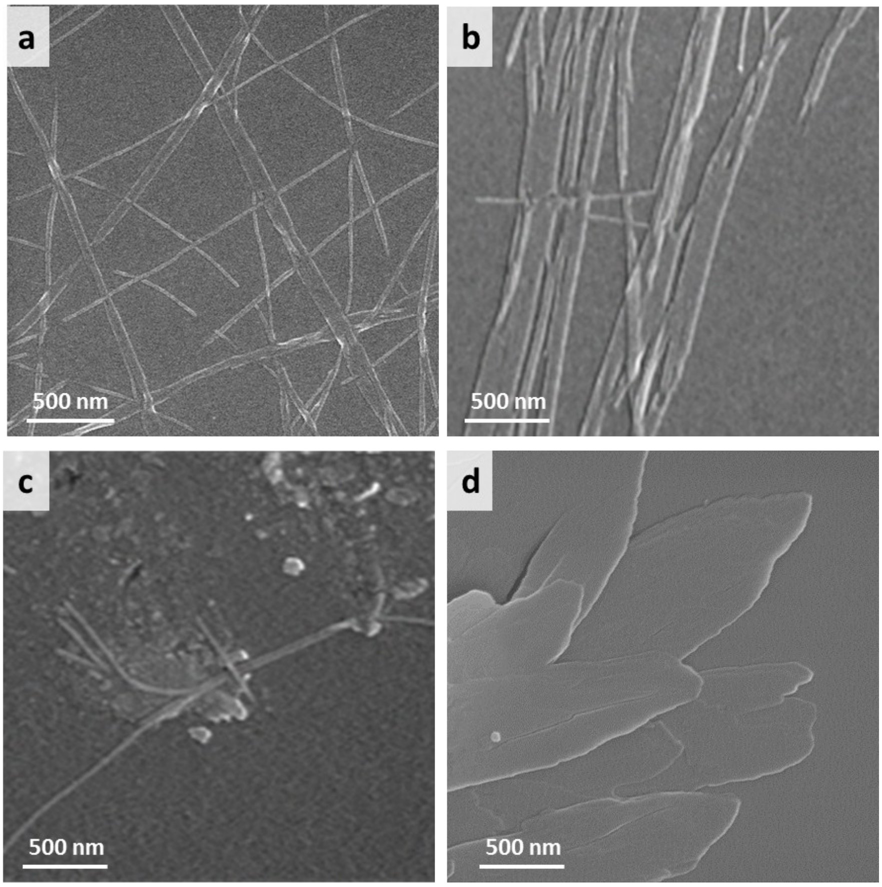

Figure S1. SEM images of **gel$_1$** in EtOH and water mixture solvent. a) **gel$_1$** in EtOH/H_2O=1:2, b) **gel$_1$** in EtOH/H_2O =1:4, c) **gel$_1$** in EtOH/ H_2O =1:6, d) **gel$_1$** in H_2O.

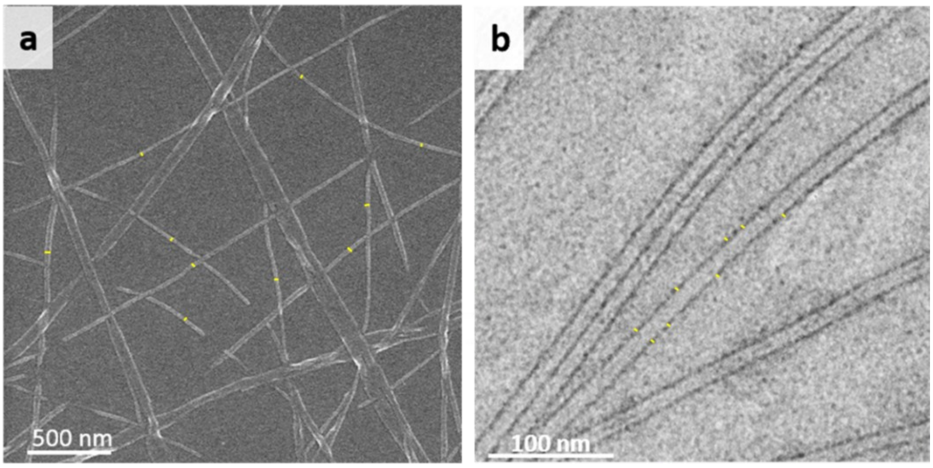

Figure S2. a) The size distribution of nanofibers in **gel$_1$**, which was about 15±1 nm; b) the size distribution of nanotubes in **gel$_2$**, which was around 2.5-2.7 nm.

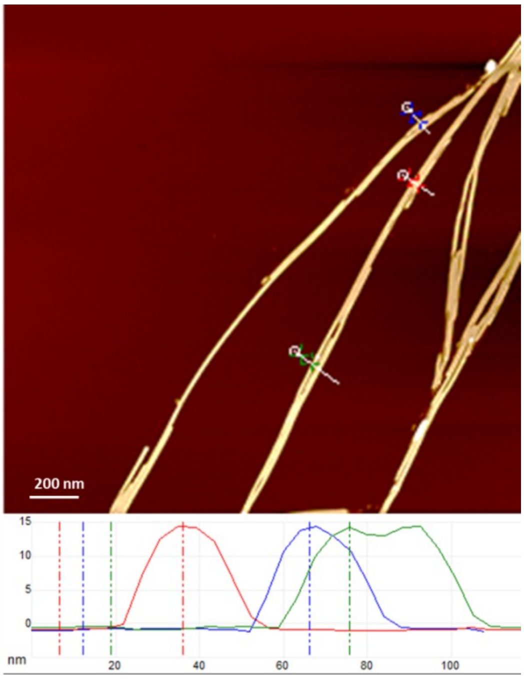

Figure S3. AFM image of gel$_1$ in ethanol water mixture solution (1:2), the height of nanofibers was about 15 nm.

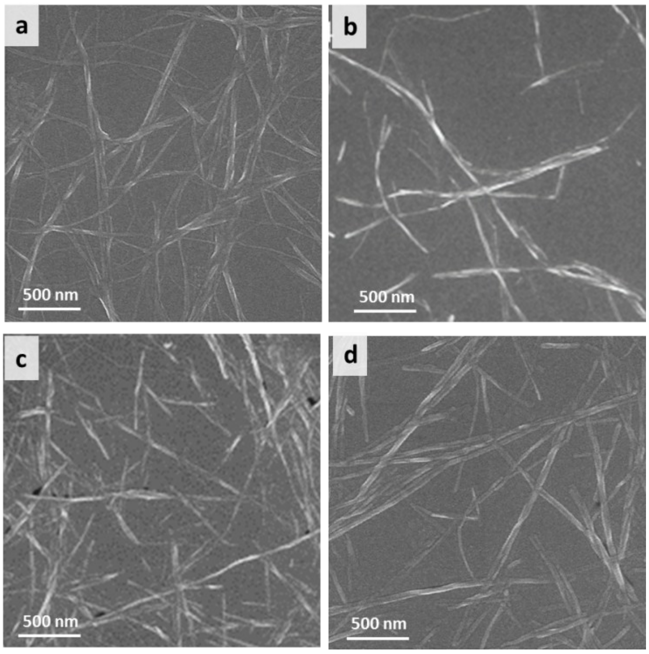

Figure S4. SEM images of **gel$_2$** in EtOH and water mixture solvent. a) **gel$_2$** in EtOH/H_2O=1:2, b) **gel$_2$** in EtOH/H_2O =1:4, c) **gel$_2$** in EtOH/ H_2O =1:6, d) **gel$_2$** in H_2O.2

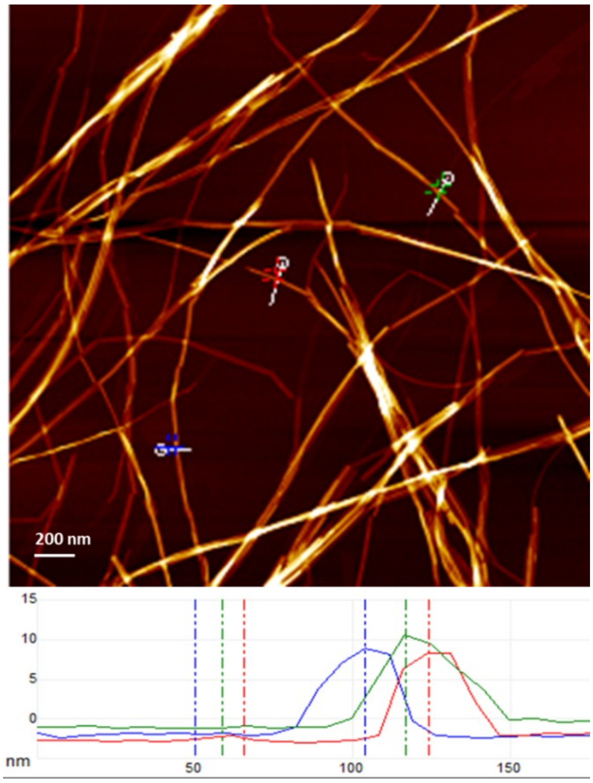

Figure S5. AFM image of **gel$_2$** in ethanol water mixture solution (1:2), the height of nanofibers was around 5-10 nm.

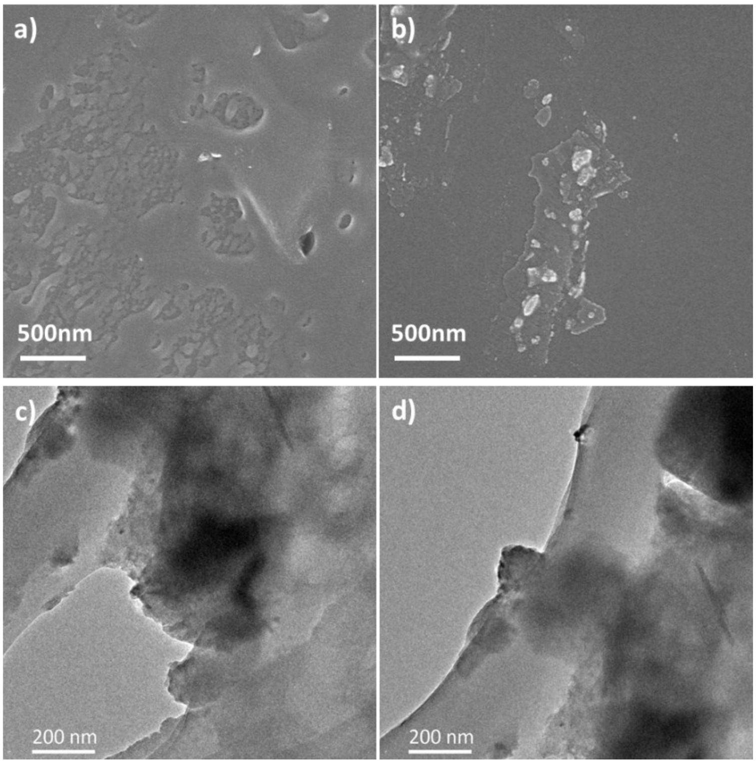

Figure S6. SEM image of a) gelator **1** and b) gelator **2** in DMSO, TEM image of a) gelator **1** and b) gelator **2** in DMSO.

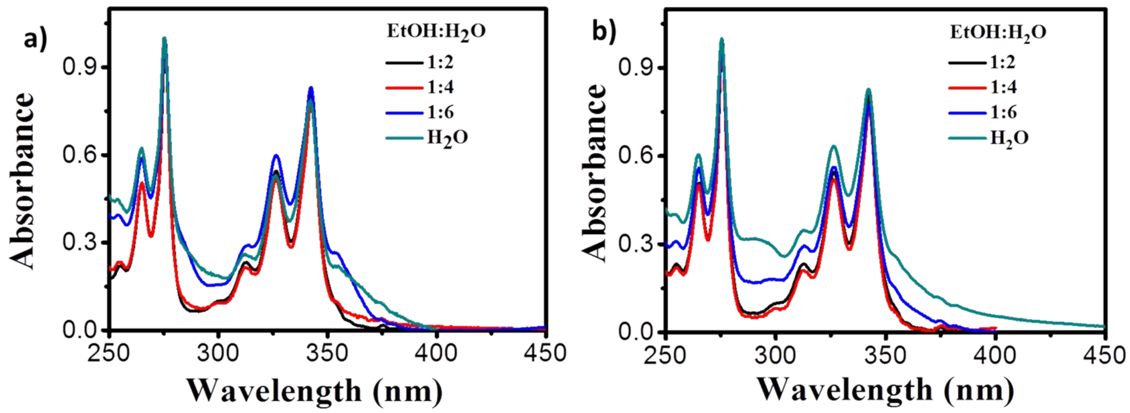

Figure S7. UV-vis spectra of a) **gel$_1$** and b) **gel$_2$** in EtOH and water mixture solvent, the absorption peaks were nearly same in the gel state.

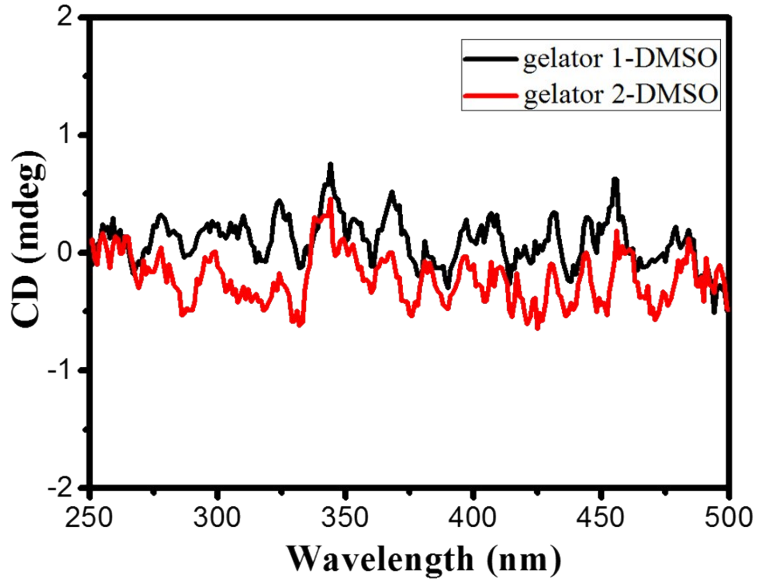

Figure S8. CD spectra of gelator **1** and **2** dissolved in DMSO.

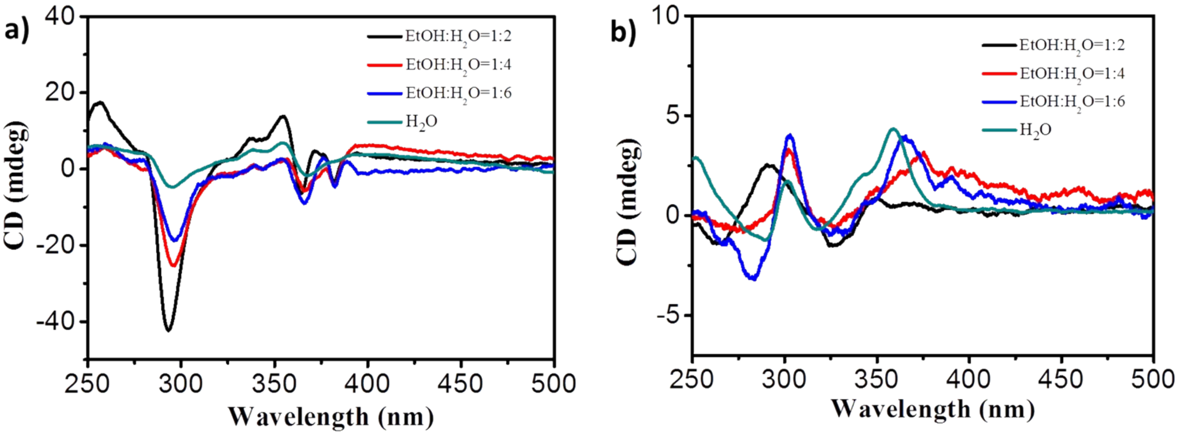

Figure S9. CD spectra of a) **gel$_1$** and b) **gel$_2$** in EtOH and water mixture solvent, all of **gel$_1$** samples under different ethanol aqueous solution present negative CD, all of **gel$_2$** samples under different ethanol aqueous solution present positive CD.

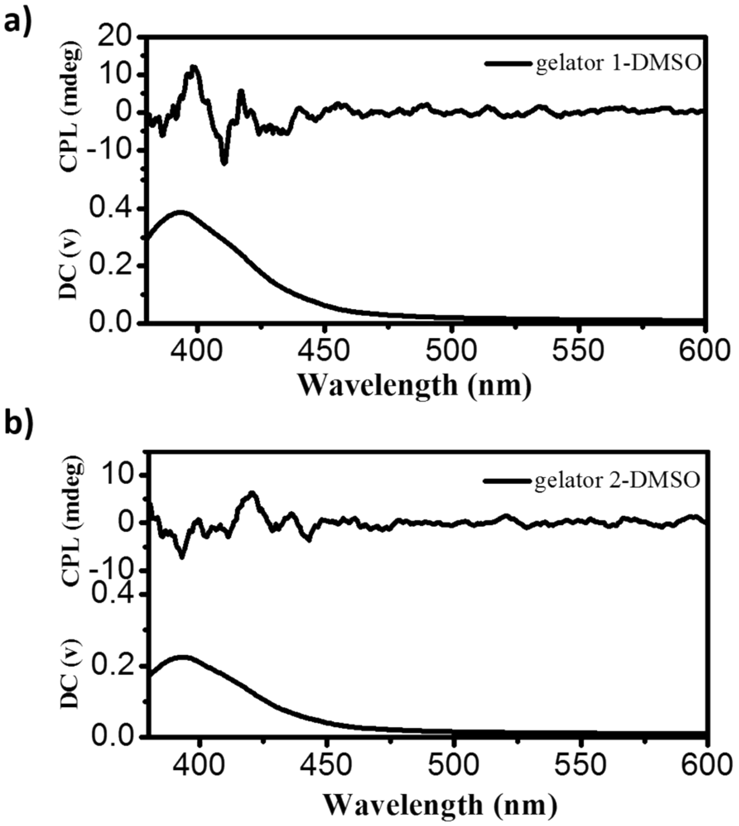

Figure S10. CPL of gelator **1** and **2** dissolved in DMSO.

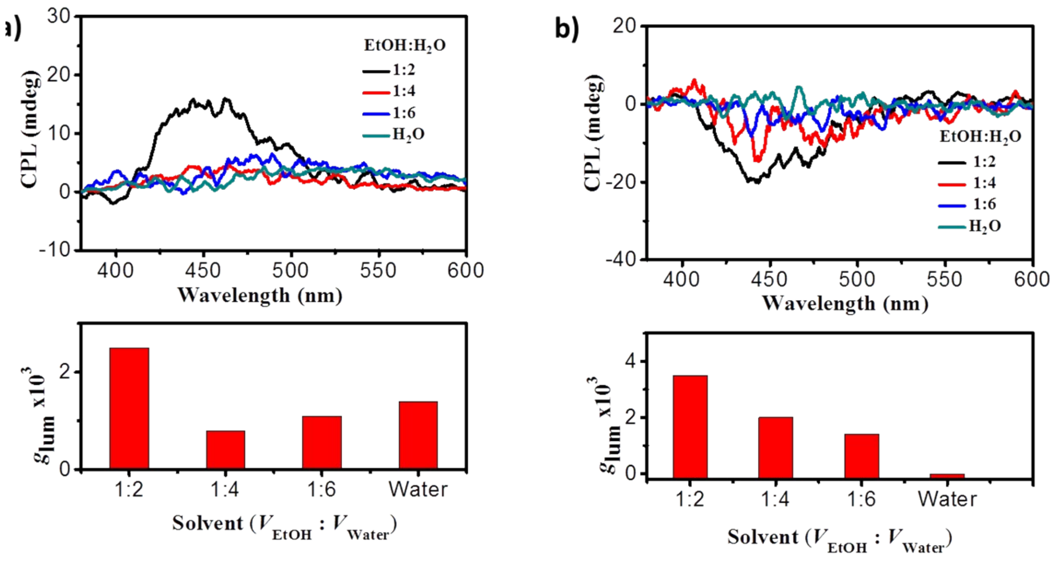

Figure S11. CPL spectra of a) **gel$_1$** and b) **gel$_2$** in EtOH and water mixture solvent, all of **gel$_1$** samples under different ethanol aqueous solution present left-handed CPL, all of **gel$_2$** samples under different ethanol aqueous solution present positive CD. The g_{lum} of **gel$_1$** and **gel$_2$** at different ethanol aqueous solvents ($V_{\text{EtOH}}/V_{\text{Water}}$), the highest g_{lum} was found at 1: 2 ethanol and water solvent both for **gel$_1$** and **gel$_2$**.
